# How the Statistical Validation of Functional Connectivity Patterns Can Prevent Erroneous Definition of Small-World Properties of a Brain Connectivity Network

**DOI:** 10.1155/2012/130985

**Published:** 2012-08-07

**Authors:** J. Toppi, F. De Vico Fallani, G. Vecchiato, A. G. Maglione, F. Cincotti, D. Mattia, S. Salinari, F. Babiloni, L. Astolfi

**Affiliations:** ^1^Department of Computer, Control, and Management Engineering, Sapienza University of Rome, 00185 Rome, Italy; ^2^Neuroelectrical Imaging and BCI Laboratory, Fondazione Santa Lucia Hospital, 00179 Rome, Italy; ^3^Department of Physiology and Pharmacology, Sapienza University of Rome, 00185 Rome, Italy; ^4^Department of Anatomy, Histology, Forensic Medicine and Orthopedics, Sapienza University of Rome, 00185 Rome, Italy

## Abstract

The application of Graph Theory to the brain connectivity patterns obtained from the analysis of neuroelectrical signals has provided an important step to the interpretation and statistical analysis of such functional networks. The properties of a network are derived from the adjacency matrix describing a connectivity pattern obtained by one of the available functional connectivity methods. However, no common procedure is currently applied for extracting the adjacency matrix from a connectivity pattern. To understand how the topographical properties of a network inferred by means of graph indices can be affected by this procedure, we compared one of the methods extensively used in Neuroscience applications (i.e. fixing the edge density) with an approach based on the statistical validation of achieved connectivity patterns. The comparison was performed on the basis of simulated data and of signals acquired on a polystyrene head used as a phantom. The results showed (i) the importance of the assessing process in discarding the occurrence of spurious links and in the definition of the real topographical properties of the network, and (ii) a dependence of the small world properties obtained for the phantom networks from the spatial correlation of the neighboring electrodes.

## 1. Introduction

The concept of brain connectivity (i.e., how the cortical areas communicate one to each other during the execution of a specific task) is central for the understanding of the organized behavior of cortical regions beyond the simple mapping of their activity [1, 2]. In the last two decades, several studies have been carried on in order to understand neuronal networks at the basis of brain processes. These networks are characterized by lots of interactions between different and differently specialized cortical sites in relation to the specific executed task. 

Cortical connectivity estimation techniques aim at describing interactions between cortical areas as connectivity patterns holding the direction and strength of the information flow between such areas. The functional connectivity between cortical areas is then defined as the temporal correlation between spatially neuronal events and it could be estimated by using different methods both in time as well as in frequency domain based on bivariate or multivariate autoregressive models [3–6] applied to hemodynamic or neuroelectrical signals. Past studies demonstrated that multivariate methods provide better estimates of connectivity patterns than bivariate approaches [7], which cannot distinguish between direct influence between two signals and the indirect common influence from a third signal [8]. For this reason, bivariate methods usually give rise to very dense patterns of propagation, thus making it impossible to find the sources of propagation [9, 10]. Different estimators, defined in time or in frequency domain and based on a bivariate or multivariate approach, rely on the concept of Granger causality between time series [11]. According to Granger's definition, an observed time series *x*(*n*) causes another series *y*(*n*) if the inclusion of *x*(*n*)'s past into an autoregressive model of *y*(*n*) significantly improves prediction of *y*(*n*). Among the more advanced estimators based on this concept, partial directed coherence (PDC) [5] is a spectral, multivariate approach allowing to describe connectivity patterns with a good accuracy and to distinguish direct from indirect information flows [5, 12]. 

The extraction of salient characteristics from brain connectivity patterns is a challenging topic, given the often complex structure of the estimated cerebral networks. For this reason, in the last ten years, a graph theoretical approach was proposed for the characterization of the topographical properties of real complex networks [13, 14]. In fact, it was demonstrated that tools already implemented and used for the treatments of graphs as mathematical objects could be applied to functional connectivity networks estimated from electroencephalographic (EEG), magnetoencephalographic (MEG), or hemodynamic (fMRI) recordings [15–19]. The use of characteristic indexes, borrowed by graph theory, allows the evaluation of real networks in terms of density of connections incoming or outcoming from a node, tendency to cluster, centrality of some nodes or edges, and distances between nodes [14, 20, 21].

 The computation of graph indexes can be performed on adjacency matrices achieved by applying a threshold on the estimated connectivity values obtained by means of different estimators. The application of a thresholding procedure allows to convert the connectivity values into edges. An edge connecting two nodes exists if the connectivity value between those nodes is above a certain threshold; otherwise the edge is null. The choice of the threshold should not depend on the application and if done in an arbitrary way could affect the results. In fact, the threshold influences the number of connections considered for the subsequent graph analysis and thus affects the indices extracted from the networks [22]. Different methodologies are available for defining such threshold. A possible approach is to select a fixed threshold. In this respect, three criteria are typically adopted: 5% significant level as a threshold fixed for discarding connectivity values from the random case [23–25]; an arbitrary value in order to discard the weak connections [26]; the largest possible threshold allowing all nodes to be connected at least to another node in the network [27]. The second way to extract a threshold is to fix the average degree within the networks in order to maximize the small-world properties of the network [28–32]. A third way to define a threshold is to fix the edge density of the network, that is, the number of existing edges divided by the number of possible edges [32]. This approach is useful if we are interested in comparing different conditions but can produce modifications in the topology of the studied network [22].

 All the approaches described above are empirical and do not take into account the intrinsic statistical significance of the estimator used in functional connectivity estimation process. In fact, when the adjacency matrix is achieved by imposing a threshold and fixing the number of residual connections of the network, we cannot exclude a priori that a percentage of such residual connections is estimated by chance. The idea is thus to take into account the statistical significance of the estimator used for functional connectivity estimation in the construction of adjacency matrix. In the case of PDC, the threshold is extracted by applying a percentile, for a defined significance level, on the distribution achieved for such estimator in the null case. Thus, an edge exists in the adjacency matrix describing the considered network only if it is statistically different from the null case.

Due to the nonlinear dependence of PDC estimator from the parameters of MVAR, the theoretical distribution of PDC in the null case in not known, so it should be constructed is an empiric way. The shuffling procedure, which has been introduced in 2001 for the similar estimator of directed transfer function (DTF) [33], allows to reconstruct the null case distribution by iterating the estimation of PDC, each time on different surrogate data sets obtained by shuffling the phases of original traces, in order to disrupt the temporal relations between them. In this way, it is possible to extract a threshold value for each couple of nodes, each direction and each frequency sample. Due to the high number of comparisons between the estimate and the null case distribution, corrections for multiple comparisons have to be taken into account. However, statistical theory offers a lot of solutions for adequately managing the occurrence of type I errors during the execution of multiple univariate tests [34, 35].

The general aim of this study is to understand how the methods for extracting the adjacency matrix could affect the graph theory indices and their interpretation, in order to define a reliable approach for the derivation of salient indices from connectivity networks estimated by means of multivariate methods. In particular, we used two different datasets with the purpose of comparing one of the methods extensively used in graph theory applications for extracting adjacency matrices from the connectivity patterns (i.e., the method based on fixing the edge density) with the statistical validation of achieved connectivity patterns by means of a shuffling procedure. The first dataset we used consisted of a set of random uncorrelated signals, which should represent a null model for functional connectivity estimates and a random case for graph theory indices. In fact, since no correlation exists between signals, the connectivity estimation process should almost entirely discard the information flows between signals, leaving only a few percentage of connections, estimated by chance and organized according to a random network. This dataset can be seen as an ideal “null case” model, but it does not take into account some factors strictly related to an electroencephalographic recording, such as the existence of a correlation between the recorded signals, due to effects of volume conduction, to the spatial positions of electrodes disposed on the scalp, and to the location of the reference [36]. For this reason, we introduced a second dataset, composed by signals recorded from a mannequin head during a pseudo experiment. This situation represents the null model for functional connectivity estimates inferred by applying partial directed coherence on EEG signals recorded at scalp level. In fact, the absence of physiological content in the recorded signals allows to model the absence of information flows between electrodes, but at the same time, the use of a real EEG cap, with electrodes positioned as 10–20 systems and references placed at the earlobes, models the effects of some factors typical of an EEG recording situation.

We estimated the functional connectivity patterns associated to both applications and we extracted the correspondent adjacency matrices by means of two approaches: fixed edge density *k* and shuffling procedure for a significance level of 5%. This second approach was explored by applying no corrections for multiple comparisons and by applying false discovery rate (FDR) correction. Several graph indexes were computed on binary adjacency matrices achieved with both methodologies. The results, achieved on the two different datasets by means of the two methods, were normalized by means of 100 random graphs with the same number of connections of the graphs obtained on simulated and mannequin data. A statistical analysis of variance (ANOVA) was performed on the results obtained by the two approaches in each dataset to study the effect of the methodology applied to the properties extracted from the networks.

## 2. Materials and Methods

### 2.1. Partial Directed Coherence

The PDC [5] is a full multivariate spectral measure, used to determine the directed influences between any given pair of signals in a multivariate data set. PDC is a frequency domain representation of the existing multivariate relationships between simultaneously analyzed time series that allows the inference of functional relationships between them. This estimator was demonstrated to be a frequency version of the concept of Granger causality [11], according to which a time series *x*[*n*] can be said to have an influence on another time series *y*[*n*] if the knowledge of past samples of *x* significantly reduces the prediction error for the present sample of *y*. In this study, the PDC technique was applied to the subset of signals *S*:

(1) $$\begin{array}{c} S={\left[s_{1}\left(t\right),s_{2}\left(t\right),\ldots ,s_{N}\left(t\right)\right]}^{T}. \end{array}$$

Let us suppose that the following MVAR process is an adequate description of the data set *S*:

(2) $$\begin{array}{c} \sum_{k=0}^{p}\Lambda_{k}S\left(t-k\right)=E\left(t\right)\;\text{with}\;\;\Lambda_{0}=I. \end{array}$$

In this expression, *E*(*t*) = [*e*_1_(*t*),*e*_2_(*t*),…,*e*_*N*_(*t*)]^*T*^ is a vector of multivariate zero-mean uncorrelated white noise process, Λ_1_, Λ_2_,…, Λ_*p*_ are the *N* × *N* matrices of model coefficients and *p *is the model order, chosen, in this case, by means of the Akaike information criteria (AIC) for MVAR processes [37]. Once an MVAR model is adequately estimated, it becomes the basis for subsequent spectral analysis. In order to investigate the spectral properties of the examined process, (2) is transformed to the frequency domain

(3) $$\begin{array}{c} \Lambda \left(f\right)S\left(f\right)=E\left(f\right), \end{array}$$

where

(4) $$\begin{array}{c} \Lambda \left(f\right)=\sum_{k=0}^{p}\Lambda_{k}e^{-j2\pi f\Delta tk}, \end{array}$$

and Δ*t* is the temporal interval between two samples.

It is then possible to define PDC as

(5) $$\begin{array}{c} \pi_{ij}\left(f\right)=\frac{\Lambda_{ij}\left(f\right)}{\sqrt{\sum_{k=1}^{N}\Lambda_{kj}\left(f\right)\Lambda_{kj}^{*}\left(f\right)}}. \end{array}$$

Such formulation was derived by the well-known concept of partial coherence [5]. The PDC from *j* to *i*, *π*_*ij*_(*f*) describes the directional flow of information from the signal *s*_*j*_(*n*) to *s*_*i*_(*n*), whereupon common effects produced by other electrodes *s*_*k*_(*n*) on the latter are subtracted leaving only a description that is exclusive from *s*_*j*_(*n*) to *s*_*i*_(*n*).

PDC values are in the interval [0,1] and the normalization condition

(6) $$\begin{array}{c} \sum_{n=1}^{N}{\left|\pi_{nj}(f)\right|}^{2} \end{array}$$

is verified. According to this condition, *π*_*ij*_(*f*) represents the fraction of the time evolution of electrode *j* directed to electrode *i*, as compared to all of *j*'s interactions to other electrodes.

Even if this formulation derived directly from information theory, the original definition was modified in order to give a better physiological interpretation to the estimation results achieved on electrophysiological data. In particular, two modifications have been proposed. First, a new type of normalization, already used for another connectivity estimator such as directed transfer function [4] was introduced by dividing each estimated value of PDC for the root squared sums of all elements of the relative row, then a squared version of the PDC was introduced [38]:

(7) $$\begin{array}{c} {\text{sPDC}}_{ij}\left(f\right)=\frac{{\left|\Lambda_{ij}\left(f\right)\right|}^{2}}{\sum_{m=1}^{N}{\left|\Lambda_{im}\left(f\right)\right|}^{2}}. \end{array}$$

The better performances of sPDC have been demonstrated in simulation studies which revealed reduced error levels both in the estimation of connectivity patterns on data characterized by different lengths and SNR and in distinction between direct and indirect paths [38]. Such formulation was used in this study for the estimation of functional connectivity.

### 2.2. Statistical Validation of Connectivity Patterns

Random correlation between signals induced by environmental noise or by chance can lead to the presence of spurious links in the connectivity estimation process. To assess the significance of the estimated patterns, each value of functional connectivity has to be statistically compared with a threshold level which is related to the lack of transmission between the considered signals at a certain probability. A possible procedure is to generate an empirical distribution of the null case based on the generation of sets of surrogate data [39] with the same spectral properties of the original dataset, but with no functional connections by construction, for example, by randomly shuffling the time series of each channel. In this study, original data were transformed from the time domain to the frequency domain, by means of Fourier Transform; then, their phases were randomly shuffled without modifying their amplitude, and finally the shuffled signals were back-transformed in the time domain. This procedure is able to keep the amplitude of the power spectrum of the time series unaltered, but at the same time to disrupt any temporal correlation between signals. A model was fit to surrogate data set and connectivity estimates were derived from the model. Iterating this process many times, each time on a new surrogate data set, allowed to build an empirical distribution of the null hypothesis for the causal estimator [33]. Once obtained the empirical distribution, we assessed the significance of the estimated connectivity patterns for a given significance level. In particular, the threshold value was evaluated for each couple of signals and for each frequency by applying a percentile, corresponding to a predefined significance level of 5%, on the null case empirical distribution. Only the connections whose values exceeded the threshold were considered significant.

### 2.3. Preventing the Occurrence of Type I Errors in Validation Process

The statistical validation process has to be applied on each couple of signals for each frequency sample. This leads to the execution of a high number of simultaneous univariate statistical tests, with consequences in the occurrence of type I errors (false positives). The statistic theory provides several techniques that can be usefully applied in the context of the assessment of connectivity patterns in order to avoid the occurrence of false positives [40]. The first one, proposed by Bonferroni in 1936, is based on the consideration that if we perform *N* independent univariate tests, each with a significance probability *β*, the probability *p* that at least one of the tests is significant is given by

(8) $$\begin{array}{c} p<N\beta . \end{array}$$

This means that if *N* = 20, tests are performed with the usual probability *β* = 0.05, then on average one of them is expected to become significant, just by chance. This means that if *N* = 20, tests are performed with the usual probability *β* = 0.05, at least one of them is expected to result, significant by chance alone. So, if we want the probability *p* for which this event could occur (i.e., one result being statistically significant just by chance) to be equal to *α*, we can apply a correction to *β*. The single test will then be performed at a probability

(9) $$\begin{array}{c} \beta^{*}=\frac{\alpha }{N}. \end{array}$$

This β* is the actual probability at which the statistical tests have to be performed to conclude that all the tests are performed at level of statistical significance *α*, Bonferroni adjusted for multiple comparisons.

The Bonferroni method can be too conservative, for instance when the statistical tests are highly dependent, like in the case of physiological measurements. This may lead to an increase of Type-II errors (false negatives). To mitigate the severity of Bonferroni approach, the false discovery rate (FDR) approach was proposed [34]. Such methodology is based on the expected proportion of erroneous rejections among all rejections. Considering **V** as the number of false positives and **S** as the number of true positives, the FDR is given by

(10) $$\begin{array}{c} \text{FDR}=E\left[\frac{V}{V+S}\right]. \end{array}$$

Let *H*_1_, *H*_2_, …, *H*_*m*_ be the null hypothesis, with *m* as the number of univariate test to be performed, and **p**_1_, **p**_2_,…, **p**_**m**_ their corresponding *P* values. These values were ordered in increasing order as **P**_(1)_ ≤ **P**_(2)_ ≤ ⋯≤**P**_(**m**)_ and the value **k **was chosen as the largest *i* for which

(11) $$\begin{array}{c} P_{\left(i\right)}\leq \frac{i}{m}\alpha . \end{array}$$

At the end, the hypothesis **H**_(**i**)_ with *i* = 1,…, *k* has to be rejected. In the case of independent tests, an approximation for evaluating corrected significance level has been introduced [35]:

(12) $$\begin{array}{c} \beta^{*}=\frac{\left(m+1\right)}{2m}\alpha . \end{array}$$

### 2.4. Graph Indexes

A graph consists of a set of vertices (or nodes) and a set of edges (or connections) indicating the presence of some sort of interaction between the vertices. The adjacency matrix *A *contains the information about the connectivity structure of the graph. When a directed edge exists from the node *j* to the node *i*, the corresponding entry of the adjacency matrix is *A*_*ij*_ = 1, otherwise *A*_*ij*_ = 0. In graph theory, a path or a walk is a sequence of vertices in which from each of its vertices there is an edge to the next vertex in the sequence. Such adjacency matrix can be used for the extraction of salient information about the characteristic of the investigated network by defining several indices based on the elements of such matrix.

#### 2.4.1. Adjacency Matrix Extraction

Once the functional connectivity pattern is estimated, it is necessary to define an associated adjacency matrix for each network, on which graph theory will be applied to extract salient indices able to characterize the network properties. The generic *ij*th entry of a directed binary adjacency matrix is equal to 1 if there is a functional link directed from the *j*th to the *i*th signal and to 0 if no link exists. As explained in Section 1, the construction of an adjacency matrix can be performed by comparing each estimated connectivity value to its correspondent threshold value. In particular,

(13) $$\begin{array}{c} G_{ij}=\{\begin{array}{c} 1\to A_{ij}\geq \tau_{ij}\\ 0\to A_{ij}<\tau_{ij} \end{array}, \end{array}$$

where *G*_*ij*_ and *A*_*ij*_ represent the entry (*i*, *j*) of an adjacency matrix *G* and a connectivity matrix *A*, respectively, and *τ*_*ij*_ is the corresponding threshold. It is possible to derive the adjacency matrix simply by applying the same threshold for all the links of the network. In this case, (13) becomes

(14) $$\begin{array}{c} G_{ij}=\{\begin{array}{c} 1\to A_{ij}\geq \tau \\ 0\to A_{ij}<\tau \end{array}, \end{array}$$

where *τ* represents the threshold to be applied to all the links in the network.

Different approaches have been developed for evaluating the threshold values, as already described in Section 1. In particular, in this study, we compared a methodology extensively used in literature and a more rigorous one proposed as an alternative in this paper. According to the first approach, the threshold is selected as the value which imposes a predefined edge density *k* (i.e., a percentage number of existing connections with respect to all possible connections, given the number of nodes in the network) for the resultant adjacency matrix. In this case, the threshold is the same for all links. The second method is based on the use of the intrinsic statistical significance of the estimator used for functional connectivity estimation. The threshold is evaluated as (1 − *α*)th percentile extracted from the null case distribution of PDC estimator built by means of the shuffling procedure. *α* is the significance level imposed in the statistical test and it was set at 0,05. In this case, a statistical threshold is evaluated for each link.

### 2.5. Graph Theory Indices

Different indices can be defined on the basis of the adjacency matrix extracted from a given connectivity pattern. In this study, we evaluated the most commonly used, described as follows.

#### 2.5.1. Characteristic Path Length

The characteristic path length is the average shortest path length in the network, where the shortest path length between two nodes is the minimum number of edges that must be traversed to get from one node to another. It can be defined as follows:

(15) $$\begin{array}{c} L=\frac{1}{n}\sum_{i\in N}L_{i}=\frac{1}{n}\sum_{i\in N}\frac{\sum_{j\in N,j\neq i}d_{ij}}{n-1}, \end{array}$$

where *L*_*i*_ is the average distance between node *i* and all other nodes and *d*_*ij*_ is the distance between node *i* and node *j* [14].

#### 2.5.2. Clustering Coefficient

The clustering coefficient describes the intensity of interconnections between the neighbors of a node [41]. It is defined as the fraction of triangles around a node or the fraction of node's neighbors that are neighbors of each other. The binary directed version of clustering coefficient is defined as follows [21].

(16) C=1n∑i∈NCi=1n∑i∈Nti(kiout+kiin)(kiout+kiin−1)−2∑j∈Ngijgji,

where *t*_*i*_ represents the number of triangles involving node *i*, *k*_*i*_^in^ and *k*_*i*_^out^ are the number of incoming and outcoming edges of nodes *i*, respectively, and *g*_*ij*_ is the entry *ij* of adjacency matrix. 

#### 2.5.3. Small Worldness

A network *G* is defined as small-world network if *L*_*G*_ ≥ *L*_rand_ and *C*_*G*_ ≫ *C*_rand_ where *L*_*G*_ and *C*_*G*_ represent the characteristic path length and the clustering coefficient of a generic graph and *L*_rand_ and *C*_rand_ represent the correspondent quantities for a random graph. On the basis of this definition, a measure of small-worldness of a network can be introduced as follows:

(17) $$\begin{array}{c} S=\frac{C_{G}/C_{\text{rand}}}{L_{G}/L_{\text{rand}}}. \end{array}$$

So, a network is said to be a small world network if *S* > 1 [42].

### 2.6. Simulated Data

The first dataset we used to compare the two approaches was generated to build the null case (complete lack of correlation between the signals). To this purpose, we generated random datasets of signals with the same average amplitude and the same standard deviation of the data acquired on the mannequin head (see following paragraph for details) to avoid differences between the two datasets due to different signals amplitudes. In particular, each dataset is composed by 20 signals segmented in 50 trials of 3s each. 20 electrodes are the typical number of sensors used for connectivity measures estimated by means of multivariate method on scalp EEG signals. 

In the following, we will refer to this dataset as “simulated data”.

### 2.7. Mannequin Data

**Figure 1:**
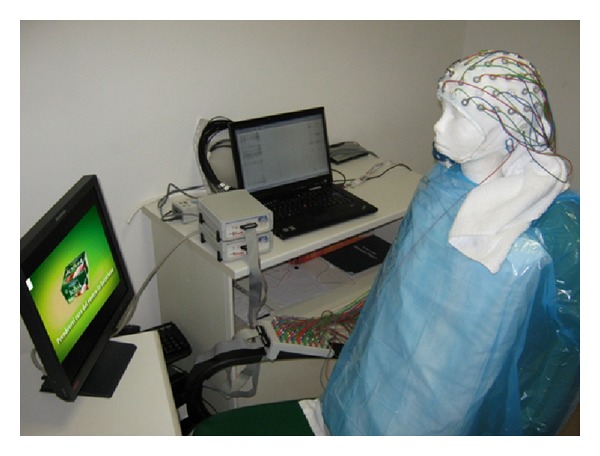
Experimental setup employed for the simulated electrical recording on a mannequin head by means of a 61-channel EEG cap. The polystyrene mannequin head was posed in front of a screen to include the interferences on signals due to the presence of a monitor.

We simulated an EEG recording on a head of a synthetic mannequin by using a 61-channel system (Brain Amp, Brain-Products GmbH, Germany). The sampling frequency was set to 200 Hz. In order to keep the impedance below the 10 kΩ, the mannequin was equipped with a cap positioned over a humidified towel. It must be noted that there were not electromagnetic sources inserted within the mannequin's head, that is instead composed only by polystyrene. Thus, the mannequin head cannot produce any possible electromagnetic signals on the electric sensors disposed on the recording cap.  Figure 1 presents the experimental setup employed for the electrical recordings. The mannequin was put in front of a screen to take into account the interferences of a monitor on EEG recording. To avoid any differences between the two datasets we used the same number of trials and samples per trial of simulated data.

We referred to this dataset as “mannequin data.”

### 2.8. Signal Processing

Both datasets were subjected to the same signal processing procedure, made by the following steps:

1. generation of 20 simulated signals (simulated data) or selection of 20 channels randomly chosen among the 61 used for the recording (mannequin data);
2. functional connectivity estimation, performed by means of sPDC;
3. extraction of the correspondent binary adjacency matrices by applying a threshold *τ* achieved in two different ways:
  - by means of shuffling procedure for a significance level of 5% in two conditions: (i) not corrected for multiple comparisons and (ii) adjusted for multiple comparisons by false discovery rate, and
  - by fixing the edge density *k* to predefined values. The levels of such values were chosen equal to those achieved by the shuffling procedure, to avoid different performances between the two methods due to the selection of a different density;
4. extraction of the graph indices described above from the adjacency matrices achieved with both methodologies;
5. normalization of the indices achieved at point 4 with those extracted from 100 random graphs generated by maintaining the same number of connections of the correspondent adjacency matrix, to normalize the values to the model dimension.

### 2.9. Analysis of Variance

The signal processing procedure (point 1 to 5 of the previous paragraph) has been repeated 50 times to increase the power of the statistical test (ANOVA) computed for comparing the two different modalities used for the extraction of the adjacency matrices.

We computed a two-way ANOVA with each graph index as dependent variable. The main factors were

- the method used for extracting adjacency matrices (METHOD), with two levels;
  - shuffling procedure,
  - fixed Edge Density procedure;
- the edge density (EDGE) corresponding to two cases:
  - Case 1: percentage of edges survived to the shuffling procedure for a significance level 5% not corrected. This percentage was resulting from the application of the shuffling procedure and was consequently imposed also to the fixed edges procedure, to avoid different performances due to different densities,
  - Case 2: percentage of edges survived to the shuffling procedure for a significance level 5% corrected by FDR. Same procedure described above.

The ANOVA was applied to both simulated and mannequin data.

## 3. Results 

### 3.1. Simulated Data

**Figure 2:**
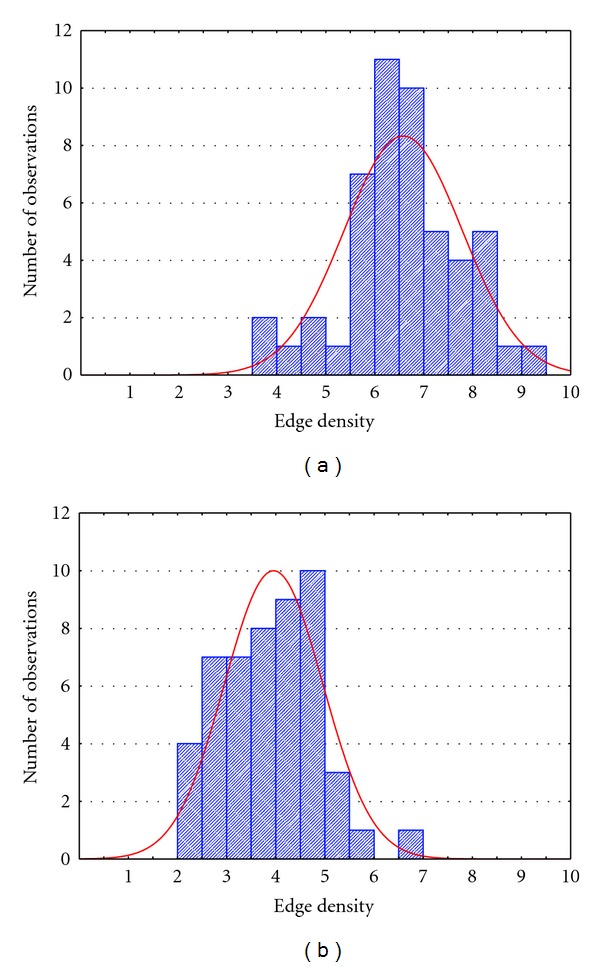
Distribution of the edge density characterizing the adjacency matrices extracted during the different iterations of the connectivity estimation process on simulated data in two different cases: Case 1 (a) → percentage of edges survived to shuffling procedure for a significance level of 5% not corrected for multiple comparison; Case 2 (b) → percentage of edges survived to shuffling procedure for a significance level of 5% corrected for multiple comparisons by means of FDR.

To describe how we selected the edge density to be used in the two approaches, we reported in Figure 2 the histograms describing the distribution of the edge density characterizing the adjacency matrices extracted during different iterations of functional connectivity estimation process on simulated data. The situations described in the two panels represent the levels Case 1 (Figure 2(a)) and Case 2 (Figure 2(b)) used for the ANOVA analysis. In particular, the average edge density resulting from the shuffling procedure applied to simulated (random uncorrelated) data was 7% for the not corrected case and 4% for the FDR corrected case. 

This first result confirmed the importance of statistical validation process combined with the correction for multiple comparisons. In fact, only the application of the shuffling procedure in the FDR case allowed to discard spurious links (obtained in this case on random, uncorrelated signals) at the correct level (below 5%). The edge densities obtained for the shuffling procedure, reported in Figure 2, were used also in the fixed edges method, to avoid different performance of the two methods to be due to the different number of connections. In particular, in the fixed edges method, if the imposed edge density is *k*, the threshold is chosen as the value which allowed to keep the *k* higher connections of the graph.

The two approaches were statistically compared by means of an ANOVA performed as described in Section 2 with each graph derived index as a dependent variable. The indices were normalized with the values obtained from 100 random graphs generated by keeping the number of connections of the correspondent adjacency matrix. This process was repeated 50 times in order to increase the robustness of the statistical analysis. 

The ANOVA analysis was computed considering the small-worldness index as dependent variable and the methods used for adjacency matrices extraction (METHOD) and the edge density of the achieved adjacency matrix (EDGE) as within main factors. The main factor METHOD was composed by two levels: shuffling procedure, fixed edge density method. The main factor EDGE was composed by two levels: Case 1 (edge density associated to significance level 5%, not corrected for multiple comparisons) and Case 2 (edge density associated to significance level 5%, FDR corrected). Results revealed a statistical influence of the main factors METHOD (*P* < 0.00001, *F* = 34.87) and METHOD × EDGE (*P* < 0.00001, *F* = 13.46) on the small-worldness index computed on connectivity networks inferred from simulated data. 

**Figure 3:**
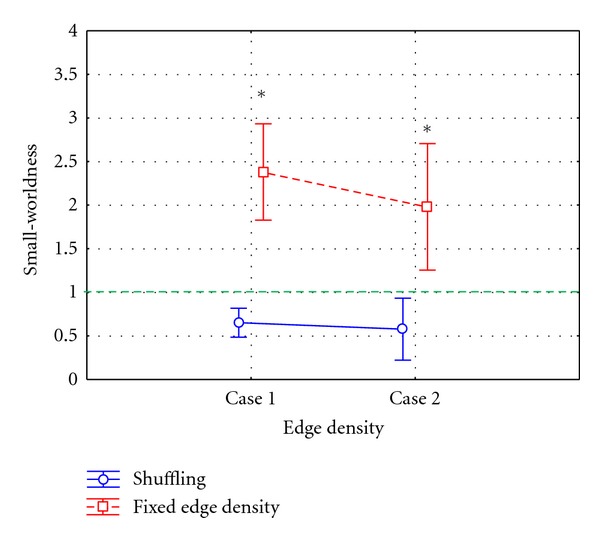
Results of ANOVA performed on the small-world index computed on networks inferred from simulated data, using METHOD and EDGE as within main factors. The diagram shows the mean value for the small-worldness computed on the adjacency matrices extracted by means of the shuffling procedure (blue line) and fixed edge density method (red line) in Case 1 (edge density as described in Figure 2(a)) and Case 2 (edge density as described in Figure 2(b)). The bars represent their relative 95% confidence intervals. The green dotted line represents the threshold above which a network is said to be “small world.” The symbol (∗) indicates a statistical difference between shuffling procedure and fixed edge density method, highlighted by Tukey's post hoc test (*P* < 0.05).

In Figure 3, we reported results of the ANOVA performed on the small world index considering METHOD × EDGE as main factor. The diagram shows the mean value for the small-worldness computed on adjacency matrices extracted by means of shuffling procedure (blue line) and fixed edge density (red line), from the connectivity patterns estimated on simulated data. The bar represented their relative 95% confidence interval. Considering that the edge density is equal by construction for the two methods, the diagram shows significant differences between the two methods in the description of the network in terms of small-worldness, confirmed by the post hoc analysis computed by means of Tukey's test (∗ symbol in Figure 3). In fact, the use of the method based on a fixed edge density revealed small-world properties of the network obtained from uncorrelated signals, for both density values. On the contrary, the application of the shuffling procedure allowed to correctly identify the absence of small-worldness in the network.

**Figure 4:**
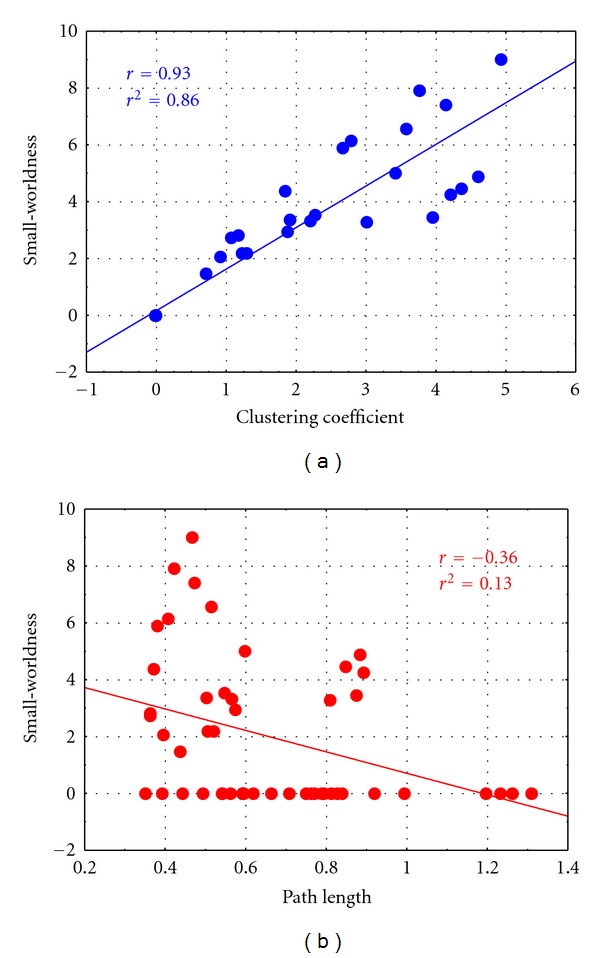
Scatterplot of small-worldness versus clustering coefficient (a) and small-worldness versus path length (b) for each iteration of the adjacency matrix extraction process computed by means of fixed edge density method for edge densities correspondent to those achieved in Case 2 (as from Figure 2(b)). The solid line represents the linear fitting computed on the data. The associated values of correlation (*r*) and *r*-square (*r* ^2^) were reported in the boxes.

To understand if the erroneous attribution of small-worldness to the networks achieved by means of the fixed edge density method is mainly due to the clustering coefficient or to the characteristic path length, correlations between the small-worldness index and these two indices were computed for the two different edge densities. The results achieved in the Case 2 (edge density as Figure 2(b)) were shown in Figure 4. The diagram showed the scatter plot of small worldness versus clustering coefficient (Figure 4(a)) and small-worldness versus path length (Figure 4(b)) for each iteration of the adjacency matrix extraction process computed by means of the fixed edge density method, in the case of edge density correspondent to those achieved in Case 2 (edge density as Figure 2(b)). The line in the figure represents the linear fitting computed on the data. In the box, the associated values of correlation (*r*) and *r*-square (*r* ^2^) were reported. From these results, it can be inferred that the small-worldness of networks achieved by means of fixed edge density method in Case 2 can be mainly due to the clustering coefficient, with a correlation of 0.93 and a *r*-square of 0.86. A minor dependence of small-worldness from path length index is highlighted by low values of correlation coefficient (−0.36) and *r*-square (0.13). The same effect can be described for Case 1 (small-worldness versus clustering *r* = 0.91, *r* ^2^ = 0.82; small-worldness versus path length *r* = −0.38, *r* ^2^ = 0.15).

### 3.2. Mannequin Data

The simulated dataset used as null model for functional connectivity estimations represents an ideal case, because it does not take into account the spatial correlation between neighboring electrodes which always occurs during an EEG recording. For this reason, we used a second dataset, composed by signals acquired simultaneously from a mannequin head equipped with a cap positioned over a humidified towel, which, with its absence of physiological signals but with its correlation between neighboring electrodes, represents the null model for connectivity inferred from signals acquired during an EEG experiment. In the second dataset, we randomly selected 20 channels among the 61 acquired (same number of signals used for simulated data) and subjected them to functional connectivity estimation process. Then the correspondent adjacency matrix was extracted by means of the two considered methods and some graph indices, such as small-worldness, path length, and clustering coefficient, were computed. The indices were normalized with the values obtained from 100 random graphs generated by keeping the number of connections of the correspondent adjacency matrix. This process was repeated 50 times in order to increase the robustness of the following statistical analysis. 

**Figure 5:**
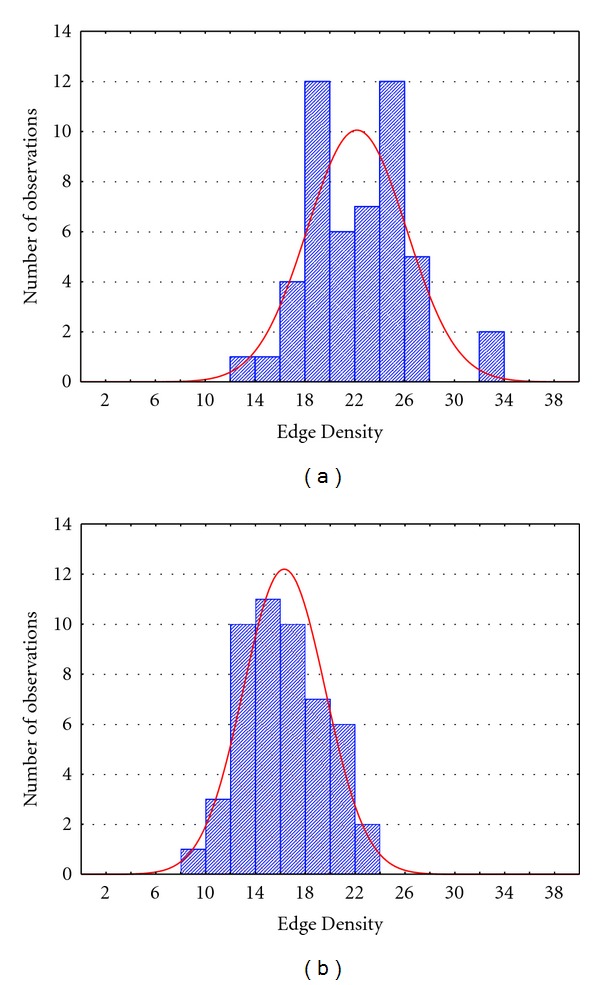
Distribution of the edge density characterizing the adjacency matrices extracted during the different iterations of connectivity estimation process on mannequin data in two different cases: Case 1 (a) → percentage of edges survived to shuffling procedure for a significance level of 5%, not corrected for multiple comparisons; Case 2 (b) → percentage of edges survived to shuffling procedure for a significance level of 5%, FDR corrected.

The shuffling procedure was applied for a significance level of 5%, both in the not corrected case and in the case of FDR correction. In Figure 5, we reported two histograms describing the distribution of the edge density characterizing the adjacency matrices extracted during the different iterations of functional connectivity estimation process on mannequin data, for the uncorrected case (Case 1, Figure 5(a)) and for the FDR corrected case (Case 2, Figure 5(b)). In particular, the average edge density was 22% for the not corrected case and 16% for the case corrected by means of FDR. This result showed the effect on connectivity measures due to the spatial correlation of neighboring electrodes. In fact, the statistical validation process combined with the correction for multiple comparisons couldn't completely discard spurious links due to random fluctuations of the signals (residual edge density above 5%). The same edge densities, reported in Figure 5, were used in the second method in order to avoid differences between the two methods due to the different number of connections. 

The same statistical analysis described in the previous paragraph for simulated data was computed on graph indices extracted from mannequin data networks. In the ANOVA, computed considering the small worldness as dependent variable and the methods use for adjacency matrices extraction (METHOD) and the edge density of the achieved adjacency matrix (EDGE) as within main factors, the main factor METHOD was composed by two levels: shuffling procedure and fixed edge density method. The main factor EDGE was composed by two levels: Case 1 (edge density as in Figure 5(a)) and Case 2 (edge density as in Figure 5(b)). Results revealed statistical influence of the main factors METHOD (*P* = 0.00001, *F* = 23.42), EDGE (*P* < 0.00001, *F* = 104.47), and METHOD × EDGE (*P* < 0.00021, *F* = 15.99) on the small-worldness index computed on connectivity networks inferred from mannequin data. 

**Figure 6:**
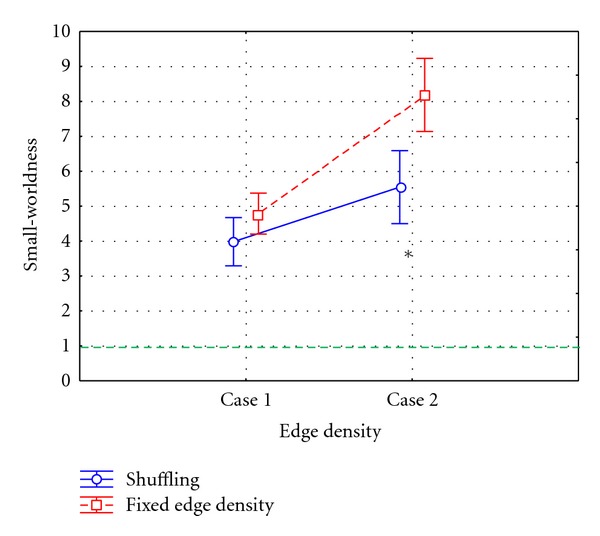
Results of ANOVA performed on the small-worldness index computed on networks inferred from mannequin data, using METHOD and EDGE as within main factors. The diagram shows the mean value for the small worldness computed on the adjacency matrices extracted by means of Shuffling procedure (blue line) and fixed edge density method (red line) in two cases, Case 1 (edge density as in Figure 5(a)) and Case 2 (edge density as in Figure 5(b)). The bar represents their relative 95% confidence interval. The green dotted line represents the threshold above which a network is said to be “small world.” The symbol (∗) indicates a statistical difference between shuffling procedure and fixed edge density method, highlighted by Tukey's post hoc test (*P* < 0.05).

In Figure 6, we reported results of the ANOVA performed on the small world index considering METHOD × EDGE as main factor. The diagram shows the mean value for the small-worldness computed on adjacency matrices extracted, by means of shuffling procedure (blue line) and fixed edge density (red line), from the connectivity patterns estimated on mannequin data. The bar represented their relative 95% confidence interval. The small-world index is above 1 for both methodologies, with statistically higher values for fixed edge density in respect to shuffling procedure in Case 2 as confirmed by the post hoc analysis computed by means of Tukey's.

**Figure 7:**
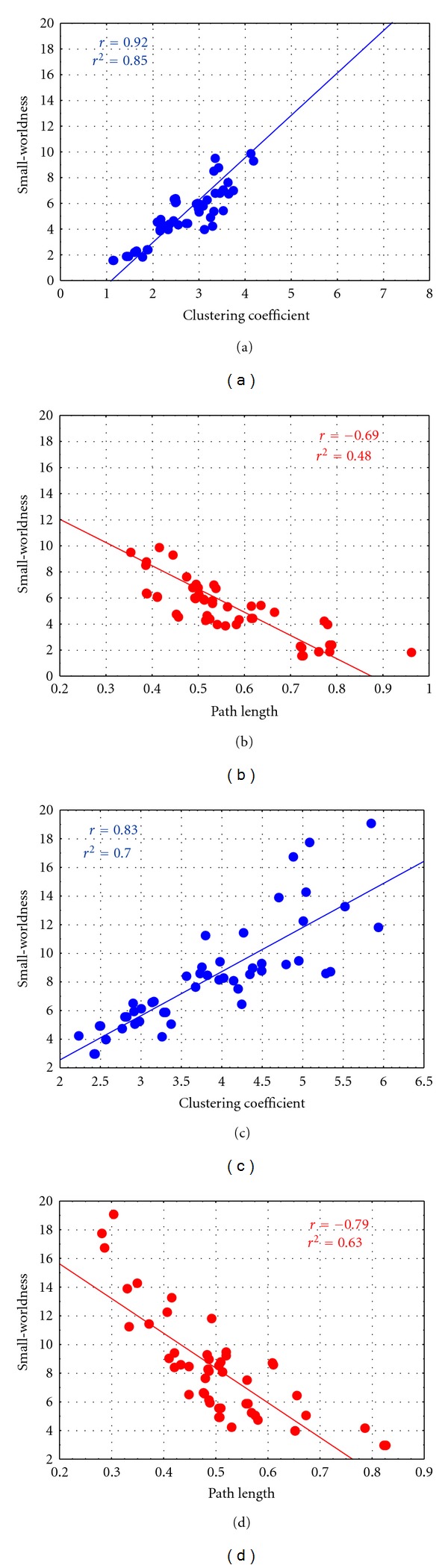
Scatterplot of small-worldness clustering coefficient ((a) and (c)) and small- worldness versus path length ((b) and (d)) for each iteration of the adjacency matrix extraction process computed by means of shuffling procedures (first row) and fixed edge density method (second row) for edge densities correspondent to those achieved in Case 2 (edge density as in Figure 5(b)). The line represents the linear fitting computed on the data. The associated values of correlation (*r*) and *r*-square (*r* ^2^) were reported in the boxes.

In order to understand which indices, between the clustering coefficient and the characteristic path length, mainly contributed to the small worldness of the networks achieved by means of shuffling procedure and fixed edge density method, correlations between the small-worldness index and these two indices were computed for the two edge density cases. The results achieved in the case of edge density correspondent to Case 2 (edge density as Figure 2(b)) were showed in Figure 7. The diagram showed the scatterplot of small-woldness versus clustering coefficient (Figures 7(a) and 7(c)) and small-worldness versus path length (Figures 7(b) and 7(d)) for each iteration of the adjacency matrix extraction process computed by means of shuffling procedure (first row) and fixed edge density method (second row) in the case of edge density correspondent to those achieved in Case 2 (edge density as Figure 5(b)). The solid lines in the figure represent the linear fitting computed on the data. In the box, the associated values of correlation (*r*) and *r*-square (*r* ^2^) were reported. The small-worldness of networks achieved by means of shuffling procedure in Case 2 can be due, at the same time, to the clustering coefficient, with a correlation of 0.92 and a *r*-square of 0.85 and to the path length with a correlation coefficient of −0.69 and a *r*-square of 0.48. Same consideration could be done for fixed edge density method (small-worldness versus clustering *r* = 0.83, *r* ^2^ = 0.70; small-worldness versus path length *r* = −0.79, *r* ^2^ = 0.63). The same effect could be described for Case 1 (shuffling procedure: small-worldness versus clustering *r* = 0.92, *r* ^2^ = 0.83; small-worldness versus Path Length *r* = −0.79, *r* ^2^ = 0.63; fixed edge density: small-worldness versus clustering *r* = 0.91, *r* ^2^ = 0.83; smal-worldness versus path length *r* = −0.87, *r* ^2^ = 0.76).

## 4. Discussion

The strong dependence of graph measures from the number of nodes, the edge density, and the degree of the networks under analysis should lead to reflect on the modalities used for adjacency matrix extraction [22]. Different methodologies are currently used for this purpose; some of them based on the definition of fixed thresholds [26, 27], others based on fixed average degree [28–32], and others on fixed edge density [32]. The choice of a threshold in order to fix the number of edges or the degree allows to avoid size and density effects in the comparison of networks inferred from two different conditions, but can affect the structure of the network by enforcing nonsignificant links and ignoring significant connections [22]. To understand the effects on the structural properties of a network due to the method applied for adjacency matrix extraction, we computed a statistical comparison between one of the methods most extensively used in graph theory applications for extracting adjacency matrices from brain connectivity patterns (i.e., the method based on fixing the edge density) with an approach based on the statistical validation of achieved connectivity patterns by means of a shuffling procedure. The comparison was performed on two different datasets, one composed by random and uncorrelated simulated data, modeling the null case for the connectivity estimates, and another one composed by signals acquired on a mannequin head, taking into account the spatial correlation between neighboring electrodes [36]. 

The results presented in this section allow to discuss about some open problems which affect the application of graph measures to the functional connectivity estimates. 

The first issue addressed in the present paper is the necessity to statistically validate the connectivity measures in order to discard the spurious links due to random fluctuations of the signals considered simultaneously in the multivariate [33, 43, 44] or bivariate model [45, 46]. In this paper, we confirmed the importance of the statistical validation combined with the corrections for multiple comparisons in multivariate estimates [47] by showing the edge densities survived to the shuffling procedure on the simulated data (Figure 2). Being the simulated data a null model for connectivity estimation, all the survived links can be seen as false positives. The application of the shuffling procedure for a significance level of 5% not corrected produces 7% of false positives. Only applying the statistical correction of FDR the false positives went down the threshold of 5%. Unfortunately, the application of shuffling procedure to connectivity networks inferred on mannequin data led to a high number of false positives (22% in the not corrected case and 16% in the case of FDR correction). This could be explained by taking into account that some of the survival links are due to real correlations between neighboring electrodes induced by the registration on a wet towel, but which can occur also in real EEG recordings [48, 49].

A second issue to be considered as relevant in graph theory concerns the modality in which the adjacency matrix is extracted from the connectivity network. As already said in the previous sections, the threshold choice is crucial for the computation of graph measures because it affects the topographical properties of real networks. In the present study, we made a comparison between one of the methods extensively used in graph theory applications for extracting adjacency matrices from the connectivity patterns (i.e., the method based on fixing the edge density) and an approach based on the statistical validation of achieved connectivity patterns by means of a shuffling procedure, to describe the effects of the modalities for adjacency matrix extraction on the “small-world” properties of the network. The results achieved on simulated data highlighted small-world properties of the analyzed networks even in random, uncorrelated data, when the fixed edge density method was applied. Such small-worldness is mainly correlated with an increase of the clustering coefficient and disappeared when shuffling procedure was used. The fixed edge density criterion led to an erroneous diagnosis of small-worldness for the connectivity patterns estimated on simulated data, independently from the edge density chosen. In fact, the simulated data, being uncorrelated, should produce connectivity patterns without any topographical properties of small-worldness. These results led to two conclusions. The first is that the shuffling procedure does not just preserve the strongest connections, as demonstrated by different results obtained by means of fixed edge density which is based on this criterion. It means that the significance of a link is not merely related to its strength. The second conclusion is that the choice of an empirical threshold can affect so much the topography of the network that an erroneous definition of small-worldness could result. Thus, a statistical validation, combined with multiple comparisons adjustments, to be applied on connectivity networks, is necessary to define the significance of each edge within the adjacency matrix, in order to extract graph measures able to describe the real properties of the considered network.

The results achieved on mannequin data showed small world properties of the networks extracted by applying both methodologies. In this case, the shuffling procedure couldn't prevent the description of mannequin networks as small world networks, even applying the corrections for multiple comparisons, but the entity of small-worldness is lower than those achieved by means of fixed edge density method. In both cases the small-worldness is equally correlated with an increase of the clustering coefficient and with a decrease of the path length. This effect could be explained with the existence of real correlations between electrodes, which can occur in real EEG data, due to volume conduction effect and to the location of the reference [36, 50, 51]. These considerations led to a possible redefinition of the meaning of the small-worldness index. In fact, it cannot be considered as an absolute measure, because its value contains some of the real correlations due to neighboring electrodes. A possible solution is to consider only variations of this measure between two conditions within the same subject, or between two subjects in the same conditions, in order to discard all the effects due to the position of the electrodes on the scalp. Another way to mitigate such effect is to apply the connectivity estimation process to the data obtained by methods which allow to reduce the spatial correlation between electrodes, such as all the approaches for the reconstruction of cortical sources from high-resolution EEG recordings [52–55]. Such methodologies allow to focus the activations of cerebral sources by means of a high number of sensors, realistic head models, and the solution of the associated linear inverse problem [56–58]. It must be also noted that other methods used for reducing spatial correlation at the scalp level, such as blind source separation and superficial laplacian [59], cannot be used, due to the correlation they induce in the data, which would, in turn, produces spurious results.

## 5. Conclusion

The present work aims at highlighting some erroneous results that can be obtained by the application of commonly used approaches for the extraction of adjacency matrix from connectivity patterns, and to describe how such procedures can affect the topographical properties of a network inferred by means of graph measures. For this reason, we computed a statistical comparison between one of the methods extensively used in graph theory applications for extracting adjacency matrices from the connectivity patterns (i.e., fixing the edge density) with an approach based on the statistical validation of achieved connectivity patterns by means of a shuffling procedure. The results achieved on simulated data highlighted the importance of a statistical validation of connectivity patterns which allows from one side to prevent the occurrence of false positives due to random fluctuations of signals, and from the other side to extract graph measures able to describe the real properties of the considered network. The results achieved on mannequin data showed an effect of the spatial correlations between electrodes and of the location of the reference on small-worldness index. Such effect could be mitigated by applying methodologies for the reconstruction of cortical sources.
